# Identifying optimal combination regimens for therapy of *Mycobacterium tuberculosis* with an algorithmic approach: prospective predictions and validations

**DOI:** 10.1371/journal.pone.0324206

**Published:** 2026-02-10

**Authors:** Arnold Louie, Michael Neely, Sarah Kim, Charles A. Scanga, JoAnne L. Flynn, Charles A. Peloquin, Brendan Prideaux, Stephan Schmidt, Mohammed Almoslem, Walter Yamada, George Drusano

**Affiliations:** 1 Institute for Therapeutic Innovation, University of Florida College of Medicine Orlando, Florida, United States of America; 2 Laboratory of Applied Pharmacokinetics and Bioinformatics, The Saban Research Institute, Children's Hospital Los Angeles, Keck School of Medicine, University of Southern California, Los Angeles, California, United States of America; 3 Department of Pharmaceutics, Center for Pharmacometrics and System Pharmacology, College of Pharmacy, University of Florida, Orlando, Florida, United States of America]; 4 Department of Microbiology and Molecular Genetics and Center for Vaccine Research, University of Pittsburgh School of Medicine, Pittsburgh, Pennsylvania, United States of America; 5 Pharmacotherapy & Translational Research, University of Florida College of Pharmacy, Gainesville, Florida, United States of America; 6 Department of Neurobiology, The University of Texas Medical Branch Galveston, Galveston, Texas, United States of America; 7 Department of Clinical Pharmacy, University of Ha'il, Ha'il, Kingdom of Saudi Arabia; Icahn School of Medicine at Mount Sinai Department of Pharmacological Sciences, UNITED STATES OF AMERICA

## Abstract

**Background:**

*Mycobacterium tuberculosis* resistance to standard-of-care agents is increasing. It is imperative to identify new combinations that increase the rate and depth of bacterial kill, shorten therapy and also suppress resistance. There has been little prior effort to identify combination regimens that employ new or repurposed drugs in a rational way.

**Methods and Findings:**

Our group developed a pathway to combine agents to achieve this end. This pathway starts with standard baseline evaluations (e.g., MIC), leverages information from *in vitro* assessments (hollow fiber infection model), then analyzes 2-agent combinations in a 96 well quantitative culture checkerboard format (Greco URSA model with simulation). Finally, development of a high dimensional mathematical model allowed evaluation of 2- and 3-drug regimens in multiple metabolic states to draw inferences regarding combination therapies. We prospectively evaluated these regimens in animal models. We showed that a prospectively chosen regimen of pretomanid, moxifloxacin plus bedaquiline performed as predicted. In the BALB/c murine model, this regimen produced sterilization in a cohort that was held for 12 weeks after therapy cessation, as it did in the C3HeB/FeJ (“Kramnik”) murine model. Finally, this and other regimens were evaluated in a cynomolgus macaque model. The decrement of the ^18^F-deoxyglucose signal in Positron emission tomography (PET)- computed tomography (CT) evaluations was best with this regimen. Other endpoints such as necropsy score and colony counts in lung and lymph nodes also demonstrated that this regimen behaved as predicted from our pathway/algorithm.

**Conclusions:**

We conclude that this provides a way forward for the future to identify the most promising regimens to shorten therapy for tuberculosis and suppress emergence of resistance.

## Introduction

Multidrug-resistant *Mycobacterium tuberculosis* (Mtb) remains a world-wide scourge accounting for an estimated 465,000 infections and 64,600 deaths in 2019 [1,2] and INH + /- Rifampin resistance is a growing problem [3–6]. An additional 5,210 deaths were attributed to extensively-resistant Mtb [2]. It is only recently that new and repurposed agents have become available to generate novel combination drug therapies that will be efficacious against wild-type isolates as well as strains with a resistance genotype.

As some of these new agents became available, they were inserted into extant regimens in a swap-in/swap-out manner. This often did not improve outcomes [7,8]. It was our hypothesis that new regimens need to be developed *de novo*, with agents that are not influenced by resistance to older agents such as isoniazid and the rifamycins.

**Fig 1:**
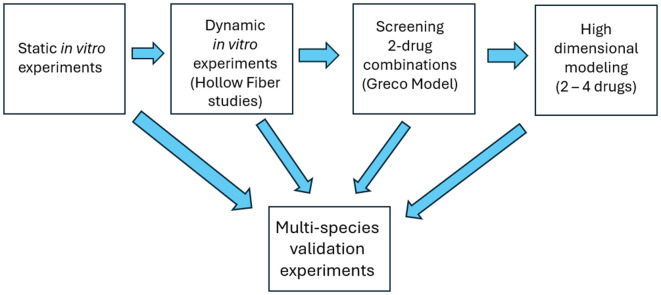
An algorithm (pathway) for identifying optimal multi-drug therapies for *Mycobacterium tuberculosis.* Citations for each shape at top for diagram: shape 2: citations 10-12; shape 3: citations 13-14; shape 4: citations 15-18.

To design novel combination therapy regimens, it is requisite to identify the desired outcomes. We sought to identify combination regimens that would be 1) unaffected by prior resistance mechanisms; 2) robust regarding suppression of resistance; and 3) highly active against Mtb in multiple metabolic states and, hence, would likely shorten the duration of therapy. The third aim is interlinked with the second aim, as a shorter regimen would lessen the likelihood that non-adherence with the treatment regimen would result in emergence of resistance [9]. Consequently, we generated and verified an algorithm (a finite set of instructions carried out in a specific order to perform a particular task – here, finding optimal combinations) or pathway for identifying optimal combination regimens using *in vitro* and animal model data in a step-wise fashion (Fig 1). The word “optimal” is meant to indicate that the identified regimen was the best drawn for the combinations studied.

Of necessity, this first step involved quantifying the activity of single agent therapy against Mtb in log phase, acid phase, and non-replicating persister (NRP) Mtb metabolic states, followed by a screening algorithm for examination of 2-drug then 3- or 4-drug regimens for the killing of Mtb in these three metabolic states. It is important to note that with six new agents entering evaluation, there are 15 and 20 possible unique combinations for 4- or 3-drug regimens. Following the existing paradigm that relies heavily on animal model data makes identifying optimal regimens a prohibitively expensive and time-consuming task. We need to be more efficient and this starts *with in vitro* evaluations to rank order possible combination regimens.

As new or repurposed agents enter the therapeutic armamentarium, often the first step, other than MIC testing or other *in vitro* evaluations such as mutational frequency to resistance and hollow fiber system evaluation, is to examine efficacy in small animal models of infection. It is critical to understand the pharmacokinetic profile (plasma and infection site) of new or repurposed agents (e.g., murine systems). These pharmacokinetic parameters are almost always quite different from that in humans (clearances tend to be higher and half-lives shorter than seen in humans). We studied linezolid in the Hollow Fiber Infection Model with murine, nonhuman primate (NHP) and human pharmacokinetic (PK) profiles [10]. For the Hollow Fiber Infection Model arms in which murine plasma PK profiles were simulated, the killing of Mtb was less than what was observed in the arms in which the NHP and human plasma PKs were simulated. Also, almost all exposures resulted in resistance emergence. Effect data should be interpreted with care when generated in small animal models.

We developed a screening methodology for ranking 2-drug combinations [11] by testing four 2-drug combinations in a 96 well quantitative culture checkerboard assay. Mtb cell kill was examined in Log-phase, Acid-phase and NRP growth. The regimens were ranked by amount of Mtb kill. The overall ranking identified pretomanid (PMD) plus moxifloxacin (MXF) as the overall best 2-drug regimen. Bedaquiline (BDQ) was identified as the agent most likely to improve the performance of the 2-drug regimen.

A full factorial design experiment with three concentrations for each single agent (n = 6) was performed with all possible 2-drug combinations (n = 9) plus a no-treatment control (total n = 16 arms) for *in vitro* Mtb killing and resistance suppression in all three metabolic states [12–14]. In addition, BDQ plus its M2 metabolite were added to the 2-drug regimens. All 16 arms were analyzed with a high dimensional mathematical model [15]. We then employed the parameter vector and full covariance matrix to simulate the estimated 95% confidence interval for kill of Mtb over time. Mtb killing was compared between the 3-drug and the 2-drug regimens. Of note, all resistant subpopulations were eradicated by week 1 in all arms.

The 3-drug regimen caused a reduction in Mtb CFU that was below the lower 95% confidence bound for 2-drug regimen by day 14, at which point the viable bacilli had reached an extinction level, indicating that the third agent (BDQ) significantly (p < 0.05) enhanced the rate and amount of Mtb killing relative to the 2-drug regimen [12–14].

The published work was performed *in vitro* and *in silico*. Here, we tested regimens predicted to be optimal in animal models of infection to **prospectively validate** the predictions.

## Materials and methods

### Antibiotics used in the *in vitro* and murine studies

PMD was graciously supplied by the Global Alliance for TB Drug Development (NY, NY). BDQ, its M2 metabolite, and MXF were purchased from BOC Sciences, Shirley, NY. Linezolid (LZD) was obtained from Curascript (Lake Mary, FL). Rifampin (RIF), isoniazid (INH), trimethoprim, cycloheximide, and polymyxin B were purchased from Millipore Sigma-Aldrich (St. Louis, MO). Pyrazinamide (PZA), piperacillin, and agarose were purchased from Thermo Fischer Scientific (Pittsburgh, PA). A suspension of 0.07% agarose, prepared in sterile water for injection, was used as the vehicle for all antibiotics that were administered to mice by oral gavage.

For the quantitative culture checkerboard studies, agar dilution susceptibility testing, and for agars supplemented with 3x the MIC of an antibiotic, the respective drugs were dissolved in DMSO and diluted to the desired concentrations in 7H9 + 10% ADC (TB broth) plus 0.02% glycerol, 0.05% Tween80 and 7H10 + 10% OADC (TB agar). The final concentration of DMSO in the TB broth and TB agar used for *in vitro* experiments was ≤ 1%.

### Microbe

Mtb strain H37Rv ATCC 27294 was purchased from the American Type Culture Collection (Manassas, VA). Stocks of the Mtb strains were frozen at -80^o^C in TB broth and 10% glycerol.

For Mtb H37Rv, log phase growth (LPG) microbes were generated by defrosting an aliquot of a frozen stock and incubating the bacteria at 35^o^C, 5% CO_2_ for 7–10 days in TB broth. Acid phase growth (APG) Mtb H37Rv was generated by transferring an aliquot of Mtb H37Rv in LPG into TB broth that was acidified to pH 6 with citric acid [16,17,18]. The pH of the medium was confirmed weekly.

Mtb isolate Erdman was kindly provided by Dr. Charles Scanga from the University of Pittsburgh. LPG and APG were generated using the same procedures that were employed with Mtb H37Rv.

*In Vitro* Methods: (i) Antibiotic susceptibility testing: Agar proportional agar MICs and microdilution broth and agar dilution MICs of antibiotics for the Mtb strains were performed as described by the Clinical and Laboratory Standards Institute [19]. MICs for APG Mtb were performed by adjusting the pH of the media to 6.0 [16,17,18]. MICs were read after the broth and agar cultures had incubated at 35^o^C, 5% CO_2_ for 2 and 3 weeks. For susceptibility studies with PMD, the bacteria inoculum was reduced to 10^4^ CFU/mL and the MICs were read after 2 weeks of incubation [19]. The MIC was read as the lowest concentration of an antibiotic that resulted in no visible growth.

To quantify the amount of BDQ and PMD that were inactivated by adding activated charcoal to TB agar, agar dilution MICs were determined for BDQ in combination with its biologically active M2 metabolite, and for LZD and PMD on TB agar, with and without the addition of 0.4% activated charcoal [20,21]. BDQ and its M2 metabolite were evaluated at a 1:4.5 ratio, which mirrors the ratio of these compounds that were measured in the ELF (epithelial lining fluid) of NHPs that were treated with BDQ [data on file, Global Alliance for TB Drug Development, New York, NY]. The Mtb inoculum was 10^4^ CFU/spot for the agar dilution susceptibility studies for BDQ and its M2 metabolite and for LZD and 10^2^ CFU/spot for studies with PMD [19]. The MICs for BDQ/M2 metabolite and LZD were read weekly after 2, 3 and 4 weeks of incubation at 35^o^C, 5% CO_2_. The MICs for PMD were read after 2 weeks of incubation. However, these agars were also read after 4 weeks of incubation to monitor for resistance selection. The highest concentration with no growth was the *minimum concentration* of BDQ/M2 metabolite, LZD, and PMD that could be inactivated when 0.4% activated charcoal was added to TB agar.

### BALB/c murine model

Six week old female BALB/c mice (mean weight of 18g) were purchased from Charles River (Wilmington, MA). On Day 0, the mice were challenged by inhaled aerosols of approximately 200 CFU of Mtb H37Rv that were grown to LPG in TB broth. The bacteria were washed by centrifugation, re-suspended in normal saline, and adjusted to 10^6^ CFU/mL by spectrophotometry. An aerosol was generated and delivered over 10 minutes using a three-jet Collison nebulizer that was controlled and monitored using an automated bioaerosol exposure system operating with a whole-body rodent exposure chamber (Biara Technologies, Hagerstown, MD) [22]. Integrated air samples were obtained from the chamber during each exposure using an all-glass impinger [23]. To verify final Mtb concentrations and exposure doses, colonies were enumerated after serial dilutions on TB agar. Five mice were sacrificed on Day 1 and at 4 weeks for quantitative cultures of the lungs and spleens to document the bacterial concentrations in these organs after bacterial challenge and at the start of antibiotic treatment, respectively. The mice were then divided into 9 groups. The no-treatment control was treated with sterile 0.07% agarose, prepared in water. Seven of the groups were treated with one-, two-, or three-drug regimens consisting of BDQ 30 mg/kg/day, PMD 100 mg/kg/day, and/or MXF 100 mg/kg/day, These combination regimens were administered as a single daily dose by oral gavage in 0.07% agarose. The ninth group served as the positive (active) control. Those mice were treated once daily with RIF 10 mg/kg/day, INH 10 mg/kg/day, and PZA 150 mg/kg/day. For combination drug regimens, the antibiotics were prepared together in agarose and administered simultaneously. The exception was the standard regimen of RIF, INH, and PZA. Grosset et al. [24] showed an antagonistic interaction between RIF and INH when they were administered together. To minimize this interaction, INH and PZA were administered together by oral gavage 0.5 to 1 hour after the mice received an oral gavage of RIF [24,25]. The dosages for the drugs were determined from *in silico* modeling of population PK analysis of plasma and lung epithelial lining fluid (ELF) PK data that were obtained from BALB/c mice after they received 7 daily administrations of antibiotic by oral gavage [10 and this publication].

Cohorts of mice are sacrificed after 1 and 2 months of treatment to characterize the extent of Mtb killing with each antibiotic regimen in the lungs and spleens of infected mice. Additional mice from each treatment arm were sacrificed 3 months after the last dose of antibiotic was administered to the mice to evaluate for sterilizing effect.

For the quantitative cultures, lungs and spleens harvested from sacrificed mice were weighed and homogenized. For treatment arms that did not include BDQ, the total Mtb burden in tissue homogenates were enumerated by quantitatively culturing the specimens on agars infused with piperacillin 50 mg/L, trimethoprim 20 mg/L, cycloheximide 50 mg/L, and polymyxin B 20 mg/L, as described by Nuremberger and colleagues [25] with modifications to prevent overgrowth of the tissue cultures with non-tubercular microbes. For mice that were treated with single and combination regimens that included BDQ, the agars used to quantitate the total Mtb population in the tissues were supplemented with 0.4% activated charcoal to mitigate the carryover effect of this drug, which accumulates within tissues [20].

The activated charcoal had the capacity to inactivate BDQ, its M2 metabolite, LZD, and PMD, as the agar dilution MICs for these drugs performed on TB agar supplemented with 0.4% activated charcoal were 26/117 mg/L (BDQ/M2 metabolite), 64 mg/L, and >128 mg/L, respectively. For agar dilution susceptibility testing performed on TB agar without activated charcoal, the MICs were 0.06/0.25 mg/L for BDQ/M2 metabolite, 1 mg/L for LZD, and 0.25 mg/L for PMD (S1 Fig).

### C3HeB/FeJ (“Kramnik”) murine TB model

The Kramnik mouse model served to evaluate the effect for Mtb in LPG, APG and NRP metabolic states on the efficacy of two combination antibiotic regimens that were evaluated in BALB/c mice [26,27].

Six week old female C3HeB/FeJ mice (Jackson Laboratory, Bar Harbor, ME) were aerosol challenged with 70 CFU/lung of H37Rv. This Mtb inoculum was achieved by shortening the 10-minute aerosol duration that was used to challenge BALB/c mice to 8 minutes. Five mice were sacrificed for quantitative cultures of the lungs and spleens on day 1 and 6 weeks after Mtb challenge to enumerate the bacterial inoculum in these organs from the aerosol challenge and at the time of initiation of antibiotic treatment. The extended incubation time relative to the 28 days used in BALB/c mice provided sufficient time for the granulomas that were produced in the tissues of Kramnik mice in response to Mtb infection to caseate [26,28]. The caseating granulomas generated a low-tension oxygen environment in which the Mtb became non-replicating persisters [27,28].

At 6 weeks of Mtb infection, the remaining mice were randomly divided into five groups. They consisted of a no-treatment control, the BPaL regimen (consisting of BDQ 30 mg/kg/day, PMD 100 mg/kg/day, and LZD 100 mg/kg/day), the 3-drug combination regimen we found to be most effective in our *in vitro* studies of PMD + MXF + BDQ [12–14] and earlier in the BALB/c TB model, the four drug regimen of PMD + MXF + LZD + BDQ that was used to determine if this combination provided more Mtb killing than the 3-drug regimens, and the standard of care (SOC) regimen of RIF + INH + PZA+Ethambutol (EMB). EMB was added to the 3-drug SOC regimen because the 3-drug SOC regimen did not provide a sterilizing effect in BALB/c mice. Comparison of the quantitative culture results of these regimens in BALB/c and Kramnik mice characterized the impact of Mtb in NRP state on the efficacies and rates of Mtb clearance by these regimens. The mice treated with the SOC regimen received RIF 0.5 to 1 hour before they were treated with INH and PZA, as described for the BALB/c mouse study. The control mice were treated once daily with 0.07% agarose. The antibiotics in the combination regimens were administered together in 0.07% agarose.

Mice from the control and treatment arms were sacrificed after 0.5, 1 and 2 months of treatment and quantitative cultures were performed on thrice-washed lung and spleen homogenates. Additional mice from each treatment arm were sacrificed 3 months after completing 1 and 2 months of antibiotic therapy to evaluate for sterilizing effect.

The quantitative cultures were performed on drug-free agar and on individual sets of agars supplemented with one of the antibiotics in the combination regimens. For regimens that included BDQ, the drug-free agars were supplemented with 0.4% activated charcoal to minimize drug-carryover. Agar containing 0.4% activated charcoal could inactivate at least 26 mg/L of BDQ, 117 mg/L of its M2 metabolite, 64 mg/L of LZD, and >128 mg/L of PMD (S1 Fig).

### Distribution of antibiotics within lungs of mice measured by matrix-assisted laser desorption ionization mass spectrometry imaging (MALDI-MSI).

#### Lung sample preparation for MALDI-MSI.

An uninfected BALB/c mouse was treated with PMD 100 mg/kg and BDQ 30 mg/kg orally every 24 hours for seven days. Five hours after the last dose was administered, the mouse was sacrificed. The lungs were resected and flash-frozen with dry ice vapors and frozen at -80^o^C until further processed for MALDI-MSI analysis.

Also, two Kramnik mice that were aerosol challenged with Mtb H37Rv 14 weeks earlier were treated with BDQ 30 mg/kg, MXF 100 mg/kg and LZD 100 mg/kg orally every 24 hours for 7 days. They were sacrificed 1 and 5 hours after the last dose was administered. The right lungs of the infected Kramnik mice were harvested, homogenized and washed thrice with sterile normal saline before they were quantitatively cultured on TB agar that was supplemented with 0.4% activated charcoal. The left lungs and spleens of the TB-infected Kramnik mice were resected and flash-frozen with dry ice vapors and stored at -80^o^C.

The tissues were processed to characterize the distribution of the antibiotics within infected organs. Infected tissues were sterilized on dry ice at UTMB using 5MRAD irradiation as previously described [29]. Fresh frozen mouse tissues were cut in 10 µm thick serial sections using a Leica CM1850 cryostat (Buffalo Grove, IL) and thaw-mounted onto Corning® alkaline earth boro-aluminosilicate glasses (CB-90IN-S111, Delta Technologies, Loveland, CO) coated with Poly-D-Lysine solution (1.0 mg/mL, A-003-E, Millipore Sigma, St. Louis, MO). The coated slides were prepared by adding enough Poly-D-Lysine solution to cover the entire surface. After 12 hours, the slides were rinsed with distillated water, dried at RT and stored at 4°C [30]. After sectioning, slides containing tissue sections were placed in a dessicator for 15 minutes. Once dessicated, the slides were placed individually in zipper sealed plastic bags and transferred to the -80C freezer for storage.

**MALDI-MSI analysis of the distribution of antibiotics in the lungs of uninfected BALB/c and in the lungs and spleens of TB-infected Kramnik mice:** Prior to MALDI-MSI analysis, the bagged slides were removed from the −80 °C freezer and allowed to reach room temperature for 15 minutes before the bags were opened. 2,4,6-Trihydroxyacetophenone (THAP) dissolved in 4:4:2 methanol/acetonitrile/water at 10 mg/ml was used as the MALDI matrix. The matrix was applied to the tissue sections by automated airspray deposition using a TM-Sprayer (HTX Technologies LLC, Chapel Hill, NC). The nozzle temperature was set to 50°C and 20 passes over the tissue were performed at a flow rate of 40 mL/min. MALDI-MSI analysis was performed using a Q-Exactive HF Hybrid quadrupole Orbitrap mass spectrometer (Thermo Fisher Scientific, Waltham, MA) equipped with a MALDI/ESI injector elevated pressure source incorporating a primary Nd:YAG laser and post ionization 266 nm solid state laser (Crylas FQSS 266–200). For MALDI-2 MSI acquisition, both the CryLas and Explorer lasers were operated at 30 Hz repetition rate, with a delay time of ~20 ns and an Explorer pulse energy of 5mJ. External mass calibration was performed using calibration solutions (A3929, Thermo Fisher Scientific, Waltham, MA). Lockmass was enabled for the protonated ion of THAP at m/z 169.04954. Spectra were acquired at 30 µm lateral resolution in positive ion acquisition mode. Total acquisition times were between 3–5 hours [31]. Thermo RAW format data files were converted to imzML using ImageInsight software (Spectroglyph LLC, Kennewick, WA). Data visualization was performed using ImageInsight or Bruker SCiLS software. Extracted ion images were plotted for LZD [M + Na]^+^ (m/z 360.133 ± 5 ppm), PMD [M + Na]^+^ (m/z 382.062 ± 5 ppm), BDQ [M + H]^+^ (m/z 555.164 ± 5 ppm), and BDQ M2 [M + H]^+^ (m/z 541.149 ± 5 ppm).

### Plasma and ELF drug concentration determinations

All concentrations were determined using validated LC-MS/MS assays on Thermo systems. RIF calibration curves were 0.25 to 50.00 µg/mL. Within-day precision was 1.08 to 4.70%CV. Overall precision (all 3 days) was 3.07 to 6.27%CV. INH calibration curves were 0.15 to 30.00 µg/mL. Within-day precision was 0.12 to 3.28%CV. Overall precision was 1.46 to 4.39%CV. PZA calibration curves were 0.50 to 100.00 µg/mL. Within-day precision was 0.36 to 2.34%CV. Overall precision was 1.66 to 3.84%CV. EMB calibration curves were 0.05 to 10.00 µg/mL. Within-day precision was 0.04 to 1.79%CV. Overall precision was 0.73 to 1.58%CV. BDQ calibration curves were 0.01 to 5.00 µg/mL. Within-day precision was 0.58 to 11.97%CV. Overall precision was 0.83 to 9.90%CV. LZD calibration curves were 0.30 to 30.00 µg/mL. Within-day precision was 0.02 to 4.42%CV. Overall precision was 1.46 to 2.91%CV. MXF calibration curves were 0.20 to 15.00 µg/mL. Within-day precision was 0.40 to 6.33%CV. Overall precision was 1.61 to 4.66%CV. PMD calibration curves were 0.01 to 5.00 µg/mL. Within-day precision was 2.06 to 9.95%CV. Overall precision was 0.72 to 7.03%CV.

### Ethics statement for murine and macaque studies

The protocols, procedures, and animal care for the BALB/c and Kramnik mice were approved by the University of Florida Institution Animal Care and Use Committee (IACUC), under IACUC protocols 201609499 and 201909499.

All protocols, procedures, and animal care for the macaques were approved by the University of Pittsburgh School of Medicine IACUC. The Animal Welfare Act (AWA) assurance number for our IACUC is A3187-01 and the U.S. Public Health Service (PHS) Assurance number is D16-00118. The specific protocol approved for this project was 19085768. The IACUC adheres to national guidelines established in the AWA (7 U.S.C. Sections 2131–2159) and the Guide for the Care and Use of Laboratory Animals (8th Edition) as mandated by PHS Policy. The macaques used in this study were pair-housed at the University of Pittsburgh in rooms with autonomously controlled temperature, humidity, and lighting in caging at least 2 M^2^. The animal care practices and enrichment plan were detailed previously [32–34].

### Non-human primate antimicrobial efficacy study

#### Animals.

The macaque study was conducted using 56 adult cynomolgus macaques (*Macaca fasicularis*) as two cohorts of 28 animals. Valley Biosystems (West Sacramento, CA) supplied the animals for Cohort 1; Alpha Genesis (Yemassee, SC) supplied animals for Cohort 2. Both male and female macaques were used. They ranged from 4–10 years old and 5–8 kg at the start of the study.

#### Mtb challenge and monitoring of macaques.

Animals were infected via bronchoscope with Mtb (Erdman strain) at a dose of 16 bacilli (Cohort 1) or 38 bacilli (Cohort 2) as previously described [33]. Animals were monitored regularly by monitoring clinical appearance, body weight, and erythrocyte sedimentation rate. Bronchoalveolar lavages and gastric aspiration were performed at 4-week intervals to detect any culturable bacilli in the airways or stomach, respectively [34]. Serial PET/CT imaging was conducted monthly as detailed below.

#### Antibiotic treatment of infected macaques.

**Fig 2:**
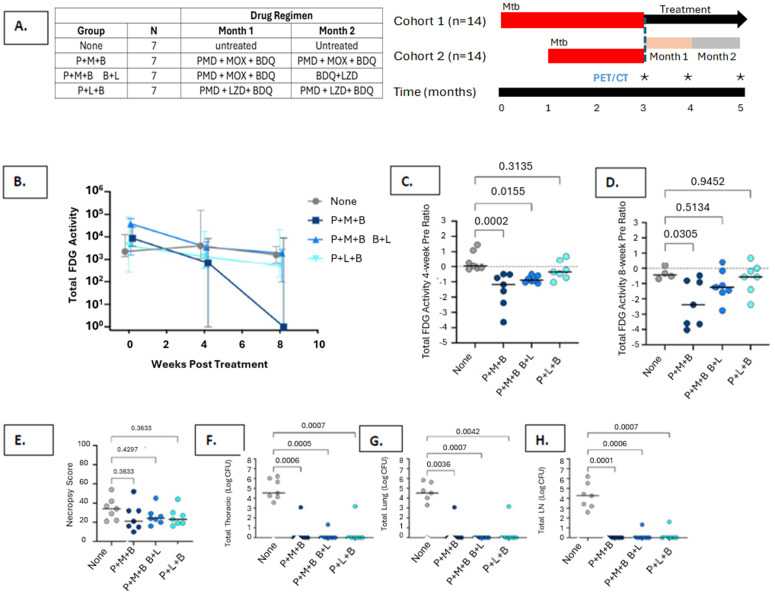
Cynomolgus macaque validation study. A. Study design; B. Overall evaluation of Total ^18^FDG Activity by Regimen; C. Comparison of Baseline Activity to Week 4 Activity by Regimen; D: Comparison of Baseline Activity to Week 8 Activity by Regimen; E: Comparison of Necropsy Score by Regimen; F. Comparison of Total Thoracic CFU by Regimen; G: Comparison of Total Lung CFU by Regimen; H: Comparison of Total Lymph Node CFU by Regimen. P = Pretomanid; M = Moxifloxacin; B = Bedaquiline; L = Linezolid.

When active disease became apparent by PET/CT imaging 8–12 weeks post infection, NHPs were randomized to a treatment group (Fig 2A). Macaques were first stratified based on disease severity and total Fluoro-deoxyglucose (FDG) activity in lungs (a surrogate for total thoracic bacterial load) [34], and then assigned to a specific treatment group, as done for similar studies [33,34]. This ensured that each experimental group consisted of animals with roughly the same range of disease. Antibiotic combinations (Fig 2A) were administered once daily by mouth by mixing into food treats. PMD was obtained from the Global Alliance for TB Drug Development (New York, NY) and dosed at 62 mg/kg. LZD was obtained from Bioduro-Sundia (San Diego, CA) and dosed at 20 mg/kg. MXF was purchased from the University of Pittsburgh Medical Center-Presbyterian Hospital pharmacy and dosed at 20.3 mg/kg. BDQ was synthesized by BOC Sciences (Shirley, NY) and dosed at 71 mg/kg. Medication compliancy was recorded daily by direct observation of the medicated treat consumption. Drugs were administered for 8 weeks. Antibiotics were discontinued at least 24 hours prior to necropsy.

#### PET/CT Imaging of lungs of macaques.

PET with CT imaging was performed using the radioprobe 2-deoxy-2-[18F]-D-deoxyglucose (FDG) at monthly intervals following Mtb infection, as previously described [32] to monitor TB progression and changes during drug treatment. A LFER-150 scanner (Mediso Medical Imaging Systems, Budapest, Hungary) and OsiriX MD (v12.0.3) medical image viewer software was used. Total FDG activity of the lungs was measured over the course of infection and drug treatment as previously described [32–34]. Granulomas in lungs were identified and measured, and standard uptake values (SUVR) were determined to assess metabolic activity as a surrogate for inflammation. SUVR values were normalized to muscle. SUVR and size of each lesion was compared pre-and post-drug treatment. A pre-necropsy scan was done within 2 days of each necropsy to map granulomas for harvest. Granulomas ≥1 mm were detectable.

#### Necropsy.

Total pathology in each macaque was quantified using a scoring metric that reflected numbers and sizes of lesions in the lungs, the size and degree of granulomatous disease in the mediastinal lymph nodes, and the extent of extrapulmonary spread [32]. Individual granulomas, as well lung lobes, thoracic and peripheral lymph nodes, liver, and spleen were excised. A portion of each tissue sample was also processed for histological analysis. The other portions were homogenized. An aliquot of the homogenates were stored at -80^o^C for future measure of antibiotic content. The remainder of the homogenates were plated as serial dilutions in PBS + 0.05% Tween-80 onto 7H11 agar plates and incubated at 37°C in 5% CO_2_ for 21 days before enumeration of Mtb CFU. Because of concerns of residual antimycobacterial compounds in the tissues, lung homogenates were also plated on 7H11 agar containing 0.4% activated charcoal, shown to bind at least 26 mg/L of BDQ in combination with 117 mg/L of its biologically active M2 metabolite (S1 Fig). The 1:4.5 ratio of BDQ to its M2 metabolic was the ratio measured in the lung ELF of macaques that were administered oral doses of BDQ. The charcoal-supplemented agar was shown to also inactivate at least 64 mg/L of LZD and >128 mg/L of PMD. Selected tissue homogenates were also inoculated into 7H9 liquid media, incubated for 7 days, then plated onto solid 7H11 agar plates containing 0.4% activated charcoal, which were then incubated for 21 days before enumeration of colonies.

#### Statistics for macaque study.

For PET and necropsy score data, groups were compared using a one-way ANOVA with Dunnett’s adjusted multiple comparison p-values. For the CFU data, groups were compared using a Kruskal-Wallis test with Dunn’s adjusted multiple comparison p-values reported. P-values of <0.05 were considered to be significant.

### Ranking metrics

For in vitro studies, the ranking metrics were *M. tuberculosis* cell kill and ability to suppress resistance. These were then tested in the animal models.

## Results

### MIC values

MICs for BDQ, MXF, PMD, LZD, RIF and INH were determined by broth microdilution and agar dilution antibiotic susceptibility assays for MTB H37Rv in LPG and APG (Table 1). MIC values for the Erdman strain (used in NHP evaluation) are also displayed in Table 1.

**Table 1:** MIC results for Mtb in log phase and acid phase growth. (A) Microbroth dilution and agar dilution MIC values (mg/L) for Mtb H37Rv tested at pH 7 and 6. (B) MIC values for the Erdman strain of Mtb. For individual Mtb strains, the same MIC values were obtained in broth and on agar.

A. Mtb H37Rv	pH 7	pH 6
PMD	0.06	0.125
MXF	0.25	0.5
BDQ	0.06	0.06
LZD	1.00	1.00
RIF	0.06	0.125
INH	0.06	0.125
B. Mtb Erdman	pH 7	pH 6
PMD	0.5*	0.25*
MXF	0.5	0.25
BDQ	0.25	0.25
LZD	1.0	1.0

### Activity of multiple anti-tubercular drug regimens in a BALB/c mouse model of TB

The use of the BALB/c murine model is a standard for evaluation of new or repurposed agents for therapy of Mtb both as single and combination regimens [21,24,25]. Mtb H37Rv exists in LPG and APG metabolic states in this mouse strain [26,27]. True caseating granulomas do not form in BALB/c mice and the pathology is granulomatous inflammation with clusters of cells often without organized structure and no caseous necrosis. Thus, Mtb do not develop an NRP state in these mice [26,27].

**Fig 3:**
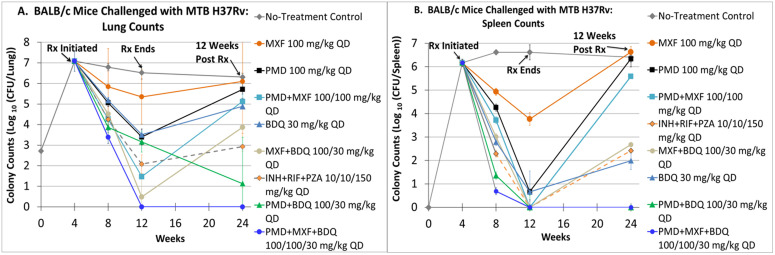
Evaluation of the bactericidal effect of single-drug, 2-drug and 3-drug combinations in BALB/c mice aerosol challenged with Mtb H37Rv. Treatment started 4 weeks after Mtb challenge and lasted for 8 weeks. Cohorts of mice were held for 12 weeks after completing 8 weeks of therapy to evaluate for sterilizing effect. A. Lung values. B. Spleen values. The combination of BDQ + PMD + LZD is not shown because the quantitative cultures were overgrown with an Enterobacter species.

We tested the efficacy of PMD, MXF and BDQ as single agents, as all possible 2-drug regimens and as a 3-drug regimen for 8 weeks (Fig 3). Controls were either no treatment (NTC) or a SOC regimen of INH, RIF and PZA. The combination of BDQ + PMD + LZD was included as a comparator. This was included because our early studies indicated that LZD had excellent activity against NRP-phase organisms. We hoped to see more rapid clearing, as we hypothesized that time to sterilization would be influenced by kill of this population. However, the quantitative cultures for this arm were overgrown by an Enterobacter species, which prevented the enumeration of Mtb colonies for that arm. A subset of animals from each experimental arm was held for 3 months after therapy discontinuation to test for sterilizing effect.

After two months of therapy, PMD and BDQ as single agents had quantitative cultures in the lungs that were 3.12 Log_10_ and 3.01 Log_10_ below that of NTC, while MXF was 1.17 Log_10_(CFU/g) below NTC (Fig 3A). For the 2-drug combinations, the declines were 5.05 Log_10_ (PMD + MXF), 3.36 Log_10_ (PMD + BDQ) and 6.04 Log_10_ (MXF + BDQ) relative to NTC. The 3-drug combination of PMD + MXF + BDQ had no recovered colonies (6.52 Log_10_(CFU/g) decline). The INH + Rif + PZA SOC had a 4.44 Log_10_(CFU/g) decline.

The story changed substantially when the cohort off therapy for 3 months was examined. For PMD, the lungs of 8/8 mice were culture positive; for MXF, 6/6 lungs were culture positive; for BDQ, 6/8 were culture positive. For the 2-drug combinations, PMD + MFX had 8/8 lungs of mice that were culture positive; MFX + BDQ had 7/8 positive; PMD + BDQ had 1/8 positive. For the 3-drug regimens, INH + RIF + PZA had 6/8 positive; PMD + MXF + BDQ had 0/8 positive.

For the spleen (Fig 3B), the relative rankings were almost the same as in the lung after 2 months of therapy for the single agents. PMD + MXF, PMD + BDQ and MXF + BDQ as well as SOC showed declines relative to NTC that were complete (sterilized). The same was true for the 3-drug regimen. This may be due to somewhat lower burdens seen in the spleen versus the lung.

The story for the spleens again changed when cohorts off therapy for three months were examined for sterilizing effect. For the single agents, PMD had 4/4 spleens that were culture positive; MXF had 3/3 culture positive; BDQ had 2/4 culture positive. For the 2-drug combinations, PMD + MFX had 4/4 positive; MFX + BDQ had 3/4 positive; PMD + BDQ had 1/4 positive. For the 3-drug regimens, INH + RIF + PZA had 2/4 positive, as in the lungs; PMD + MXF + BDQ had 0/4 spleens that were culture positive.

The results with the 2-drug regimens are consistent with the hypothesis that an extremely slow rate of egress of BDQ from the anatomical site may explain the rankings for both BDQ-containing regimens. It should also be noted that in the spleen, there were 2 regimens that had no recoverable counts at 3 months off therapy, PMD + BDQ and PMD + MXF + BDQ. It is important to note that these samples were cultured on agar containing 0.4% charcoal to absorb residual drug.

### Mtb isolates cultured from BALB/c mice with reduced susceptibilities to an antibiotic

The homogenates of lungs and spleens collected from mice in the control arm and from the PMD monotherapy arm had colonies which grew on TB agar supplemented with 3x MIC of PMD. The control arm also had colonies which grew on TB agar supplemented with 3x MIC of BDQ. The colonies that grew on the PMD supplemented agars in the control and PMD monotherapy arms had PMD MICs of 1 mg/L. The parent isolate had a PMD MIC of 0.125 mg/L.

None of the other treatment arms had growth of Mtb colonies on antibiotic-supplemented agars. BDQ monotherapy was sufficient to prevent the selection of colonies with reduced susceptibilities to BDQ. It may be that the selection pressure of 3 x MIC in the resistance plates was too great as it has been shown that some mutations provide low grade BDQ resistance [35,36]. PMD, in combination with BDQ or MXF, effectively counter-selected for PMD resistance.

### Activity of multiple anti-tubercular drug regimens in a Kramnik mouse model of TB

C3HeB/FeJ (“Kramnik”) mice develop caseating granulomas in response to Mtb infection [26]. The caseating granulomas provide a hypoxic environment that promotes the transition of Mtb to an NRP metabolic state [28]. Hence, Mtb in Kramnik mice exist in LPG, APG, and NRP metabolic states [26,27].

**Fig 4:**
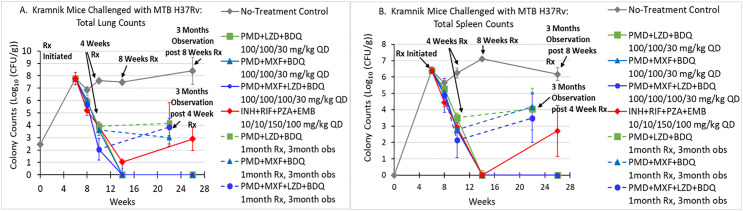
Evaluation of the bactericidal effect of 3- and 4-drug combinations in C3HeB/FeJ (Kramnik) challenged with Mtb H37Rv. Treatment started 6 weeks after Mtb challenged and lasted for up to 8 weeks. Cohorts of mice that completed 4 or 8 weeks of therapy were observed for 3 months to evaluate for sterilizing effect. A. Lung values. B. Spleen values.

In the Kramnik mouse Mtb model, two 3-drug regimens, a 4-drug BDQ-containing regimen, a 4-drug SOC regimen consisting of RIF + INH + PZA + EMB, and a no-treatment control (NTC) were evaluated. The results are displayed in Fig 4A (lung) and 4B (spleen).

Efficacy was evaluated immediately after completing 0.5, 1 and 2 months of therapy to evaluate for rates of Mtb killing and bactericidal effect. Additional evaluations were made 3 months after Kramnik mice had completed 1 and 2 months of therapy to assess for sterilizing effect. Evaluation for sterilizing effect was not performed for animals that received 1 month of the SOC regimen because that regimen did not have sterilizing effect in the BALB/c mouse study described earlier.

In the lungs, all the antibiotic regimens, including the SOC, produced similar amounts of killing of Mtb after 0.5 months of treatment. At 1 month of treatment the 4-drug regimen of BDQ + PMD + LZD + MXF reduced burden by 2.06 ± 1.41 log_10_(CFU/g) of lung compared to 3.91 ± 0.18 log_10_(CFU/g) and 3.64 ± 0.47 of lung for BDQ + PMD + LZD and BDQ + PMD + MXF, respectively. The difference in quantitative cultures between BDQ + PMD + LZD and BDQ + PMD + MXF after 1 month of treatment was not significantly different (*p* = 0.40). But the difference between the quantitative culture values for the 3-drug regimens and for BDQ + PMD + LZD + MXF was significantly different (*p* = 0.02 and 0.038, respectively). With 1 month of treatment, none of the 3- and 4-drug regimens containing PMD + BDQ had sterilizing effects (Fig 4A). Mtb was not cultured from the lungs of mice treated with PMD + LZD + BDQ, PMD + MXF + BDQ, and PMD + MXF + LZD + BDQ after 2 months of therapy. Also, Mtb was not recovered in cultures performed on lungs harvested from mice 3 months after the completion of 2 months of antibiotic therapy, showing the 3 regimens had sterilizing effect. In contrast, animals receiving the SOC regimen had 1.33 Log10(CFU/g) at 8 weeks of treatment and all of the SOC mice had positive cultures at 3 months after stopping therapy. The NTC group had Mtb burdens in the lungs of 7.60 and 8.13 Log_10_(CFU/g) at these time points (all were positive).

In the spleens, the efficacy of the three PMD + BDQ-containing regimens after 0.5, 1 and 2 months of therapy were similar (Fig 4B). These regimens did not have a sterilizing effect in the spleens after 1 month of treatment. After 2 months of treatment, these regimens yielded negative splenic cultures and the three regimens had a sterilizing effect. The SOC produced a similar amount of Mtb killing in the spleen as the other treatment regimens at the 0.5, 1, and 2 month treatment time points. This included undetectable Mtb counts at the 2 month treatment time point. However, the cultures of spleens were positive in all SOC mice that were sacrificed 3 months after they completed 2 months of treatment, showing 2 months of treatment the 4-drug SOC regimen did not have sterilizing effect in the spleens.

### Mtb isolates cultured from Kramnik mice with reduced susceptibilities to an antibiotic

The homogenates of lungs and spleens collected from mice in the NTC arm had bacilli which grew on TB agar supplemented with 3x MIC of PMD and had PMD MICs of ≥ 1 mg/L. For comparison, the Mtb isolate used for infection exhibited a PMD MIC of 0.125 mg/L.

None of the lung or spleen samples of mice treated with BDQ + PMD + MXF, BDQ + PMD + LZD, and the SOC regimen of RIF + INH + PZA grew Mtb when cultured on agar supplemented with 3x MIC of one of the antibiotics administered to that treatment arm.

### Activity of different treatment regimens in cynomolgus macaques

Drug combinations were tested in cynomolgus macaques intrabronchially infected with a virulent strain (Erdman) of Mtb. Macaques developed active TB disease 2–3 months after infection, as determined by ^18^FDG PET/CT imaging and other clinical parameters. Antibiotic drug regimens were initiated once active TB was determined. The experimental groups each consisted of 7 monkeys, which were either untreated or treated with PMD + MXF + BDQ for 2 months, PMD + MXF + BDQ for 1 month followed by LZD + BDQ for an additional month, or PMD + LZD + BDQ for 2 months (Fig 2A). TB disease was monitored by FDG PET/CT imaging performed every 4 weeks. Two months after treatment initiation, macaques were subjected to a detailed necropsy as previously described [32–34].

Consumption of the drug doses was directly observed and recorded by experienced veterinary technicians. All animals reliably consumed their drug-laced treats throughout the treatment course. Compliance and drug absorption were further confirmed by measuring drug concentration in blood drawn every two weeks during the treatment course (not shown).

FDG PET/CT imaging has been shown by us and others, in humans and in macaques, to identify successful response to drug treatment, reflected by reductions in inflammation in the lungs [32–34]. In untreated animals, there was no reduction in FDG activity in lungs over 2 months (Fig 2B). Treatment with the PMD + MFX + BDQ regimen for 2 months resulted in substantial reduction in FDG lung activity, while the PMD + MXF + BDQ/LZD + BDQ and PMD + LZD + BDQ regimens showed only modest reductions. Measuring FDG activity after 1 month of treatment (Fig 2C) showed that PMD + MFX + BDQ for just 1 month significantly reduced FDG activity compared to pretreatment values in both the PMD + MXF + BDQ and PMD + MXF + BDQ/LZD + BDQ groups (p = 0.0002 and 0.0155, respectively, compared to untreated group). It should be noted that antibiotics in these regimens were identical at the one month time point (the switch had not occurred). The group treated with PMD + LZD + BDQ exhibited no reduction in FDG activity after 1 month (p = 0.3135 vs untreated group) (Fig 2C). Continuing the PMD + MXF + BDQ regimen for a second month of treatment resulted in a further reduction in FDG lung activity (p = 0.0306 vs untreated macaques) (Fig 2D). However, switching macaques from PMD + MFX + BDQ to LZD + BDQ for the second month of treatment did not result in additional reduction in FDG lung activity (p = 0.5154 vs untreated) (Fig 2D). PMD + LZD + BDQ for 2 months did not reduce FDG lung activity. Thus, using PET/CT measures as a marker of drug treatment, the most successful regimen was the 2 month PMD + MFX + BDQ regimen.

Total pathology at necropsy was assessed by the University of Pittsburgh quantitative necropsy score metric [32] and did not reveal significant differences in pathology between any groups (Fig 2E). This was not surprising as this score takes into account all lesions found in lungs, lymph nodes, or extra-pulmonary sites. Our previous data support that the lesions (e.g., granulomas and other pathologies) can still be observed even with successful drug treatment [32–34]. Interestingly, nearly all macaques in the three treatment groups were sterile at necropsy and all had significantly reduced total thoracic, lung, and lymph node bacterial burden compared to untreated macaques (Fig 2F–2H). This was in contrast to PET data which indicated that the PMD + MXF + BDQ regimen best reduced lung inflammation (Fig 2C, 2D). To rule out that drug carryover in the tissue homogenates that were plated inhibited Mtb growth, we re-plated homogenates onto 7H11 media containing 0.4% activated charcoal shown to neutralize at least 28 mg/L BDQ and 126 mg/L of its bioactive M2 metabolite [S1 Fig], 64 mg/L of LZD, and >128 mg/L of PMD (data not shown). We also inoculated 7H9 liquid cultures from selected samples to dilute any residual drugs and cultured them for growth. None of these samples yielded culturable bacilli. Thus, after 2 months of treatment, all regimens were quite successful at sterilizing Mtb infection in macaques. However, FDG PET imaging suggests that the 2-month PMB regimen was able to resolve inflammation fastest, and perhaps induce sterility, in Mtb-infected macaques.

### Distribution of antibiotics in tissues of uninfected BALB/c mice and C3HeB/FeJ (Kramnik) mice infected with Mtb H37Rv determined by MALDI-2 MSI

The activity of antibiotics may be explained, in part, by the metabolic state of the microbe and on its distribution in different sites within tissues. It may also be determined by the speed in which the drug enters and effluxes from these sites.

**Fig 5:**
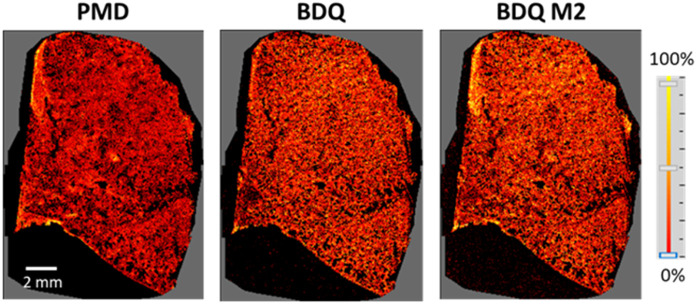
MALDI-2 MSI studies showing homogenous distribution of PMD, BDQ, and BDQ M2 metabolite in the lungs collected from an uninfected BALB/c mouse 1.5 hours after it had received daily doses of these antibiotics for seven days.

The lungs were harvested from a sacrificed, uninfected BALB/c mouse, 5 hours after the animal had received seven daily doses of orally dosed PMD and BDQ. Due to the absence of lesions or immune/inflammatory foci, PMD, BDQ, and the M2 metabolite of BDQ were homogeneously distributed throughout the lungs of this mouse (Fig 5).

Kramnik mice were infected by aerosol inhalation with Mtb H37Rv 14 weeks before they received seven daily oral doses of BDQ, MXF, and LZD. The lungs and spleens were harvested for MALDI-2 MSI analysis at 1 and 5 hours after they received the seventh dose of the antibiotics.

**Fig 6:**
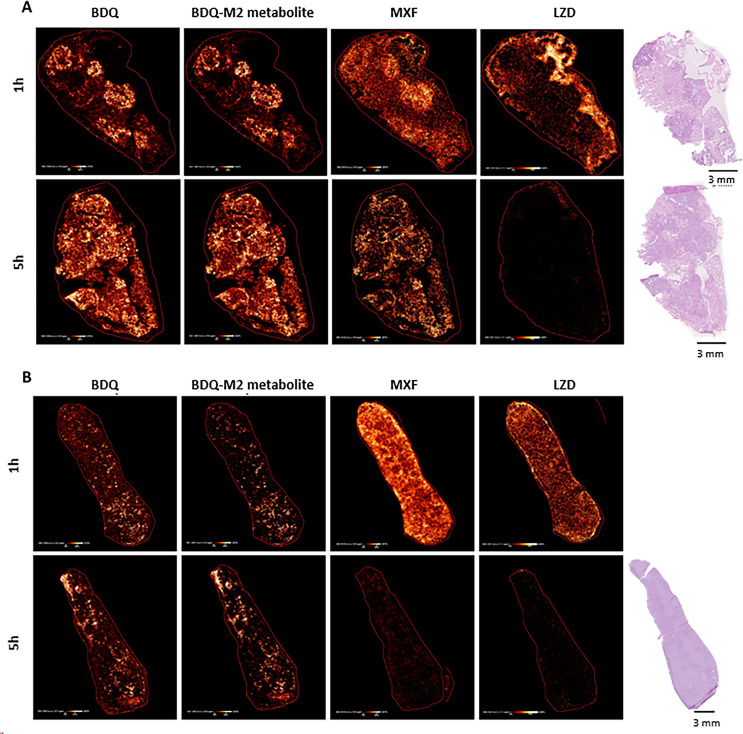
MALDI-2 MSI studies showing distribution of BDQ, BDQ M2 metabolite, MXF, and LZD in the lung (A) and spleen (B) of C3HeB/FeJ (Kramnik) mice. The organs were harvested from mice that were sacrificed 1 and 5 hours after they have received daily doses of these antibiotics for seven days. H&E stains of the lungs are also shown. The Kramnik mice were aerosol challenged with Mtb H37Rv 14 weeks prior to the initiation of antibiotic therapy.

All three drugs exhibited heterogeneous distribution throughout the lungs (Fig 6A). At the 1-hour post-final dose timepoint, the highest intensities of BDQ and its M2 metabolite were observed within granulomas, with lower drug signals detected in the surrounding parenchyma. Given the long half-life and established uptake and accumulation of BDQ and BDQ M2 in macrophages, this elevated signal in granulomas is likely attributable to macrophage-mediated drug uptake and retention from previous administration of BDQ. MXF demonstrated a similar distribution pattern, with the strongest signal also localized within granulomas. In contrast, the highest LZD signal was detected at the periphery of the tissue, co-localizing with pooled blood. Within the tissue itself, LZD showed greater signal in the parenchyma, with limited, but detectable, penetration into granulomas. At the 5-hour post-final dose timepoint, BDQ and BDQ M2 displayed a more homogeneous distribution throughout the tissue, with increased signal in the parenchyma likely reflecting drug ingress following the final dose. MXF signal remained highest within the granulomas, particularly along the lesion periphery, with evidence of egress from the parenchyma. By this timepoint, LZD was scarcely detectable within the tissue.

Drug distribution within spleen tissue was heterogeneous (Fig 6B). Both BDQ and its metabolite BDQ M2 localized to the red and white pulp, with distinct, intense foci observed in the white pulp. This distribution pattern was consistent at both 1 hour and 5 hours following the final drug administration. MXF was similarly distributed throughout the tissue at the 1-hour timepoint; however, a stronger drug signal was noted in the red pulp. By 5 hours, the drug had begun to egress, and the highest remaining signal was localized to the white pulp. The distribution of LZD in the spleen mirrored that of MXF at 1 hour. By 5 hours, minimal signal remained, with the residual drug localized primarily to the white pulp.

### Pharmacokinetics of antibiotics in mice and NHPs with tuberculosis

The plasma population pharmacokinetics and the volume of the lung epithelial lining fluid (ELF) for PMD, MXF, BDQ, and LZD in mice with tuberculosis are provided in S1–S4 Table. The plasma population pharmacokinetics and volumes of ELF for PMD, MXF, and LZD in NHPs are provided in S1, S2, and S4 Tables. A PK study was not performed for BDQ in NHPs since the Global Alliance for TB Drug Development (New York, NY) had performed that investigation previously.

### Concentrations of antibiotics in the lungs of NHPs treated antibiotics

Quantitative cultures were performed on tissue samples that were collected from the lungs of NHPs with tuberculosis who were treated with antibiotics for one or two months. The samples were collected 24 hours after the animals had received their last dose of antibiotics. The samples were homogenized. An aliquot of the homogenates was stored at -80^o^C for subsequent measure of antibiotic content by UPLC/MS/MS. The remainder of the samples were quantitatively cultured on agars infused with 0.4% activated charcoal.

A substantial majority of quantitative cultures were sterile after two months of antibiotic therapy (Fig 2F–2G). The quantitative cultures of tissues collected from the no-treatment control mice grew Mtb. We showed that the activated charcoal added to the agars was able to inactivate that least 26 mg/L of BDQ, 117 m/L of the M2 metabolite of BDQ, 64 mg/L of LZD, and >128 mg/L of PMD (S1 Fig).

To determine if the lack of growth of the quantitative cultures was due to drug carryover, the aliquots of tissues stored at -80^o^C were thawed and the concentrations of BDQ, the M2 metabolite for BDQ, LZD, MXF, and PMD in the samples were measured by UPLC/MS/MS. The results are shown in Table 2. All of the concentrations of the antibiotics measured in lung samples were below the concentrations that could be inactivated by the activated charcoal that was added to the agars used for the quantitative cultures. Hence, it is highly likely that two months of the antibiotic regimens administered to the NHPs sterilized these tissues. The absence of growth in the quantitative cultures was not due to carryover of the antibiotics in the tissues that were plated onto the agars used for performing the quantitative cultures.

**Table 2:** Antibiotic concentrations (mg/L) in samples from lungs of non-human primates sacrificed 24 hours after the last dose was administered. Samples were homogenized and then assayed for drug concentrations by UPLC/MS/MS.

	BEDAQUILINE	M2 Metabolite	LINEZOLID	MOXIFLOXACIN	PRETOMANID
No. of Samples mg/L	287	356	35	48	138
Minimum	0.001	0.003	0.0490	0.000	0.000
Maximum	18.07	84.600	0.274	0.000	0.242
Median	0.197	1.682	0.0580	0.000	0.003
Arithmetic Mean	2.406	9.172	0.0839	0.000	0.0203
Standard Deviation	3.971	14.80	0.0591	0.000	0.0453

## Discussion

Our overarching goal was to identify multi-drug regimens for the therapy of Mtb that would rapidly sterilize the infection site, thereby shorten therapy as well as minimize the likelihood of resistance emergence. Regimen shortening would also likely have a beneficial effect on resistance suppression.

To address this need, we developed *in vitro* methodologies (kill curves and Hollow Fiber Infection Model) that allowed insight into how new or repurposed agents might be ranked as single agents [10,13,14,16,37–39] and what pharmacodynamic index was most closely linked to resistance suppression.

We also developed a screening methodology to rank 2-drug combinations, based on the Greco URSA Model [11,40]. The experiment was performed in a 96 well plate quantitative culture checkerboard format with bacterial counts as the system output. The Greco model allows identification of the interaction parameter α to determine the mode of interaction of the two drugs (i.e., synergy, additivity, antagonism). The point estimate also has a 95% confidence interval, so that the statistical significance of the classification can be identified. We also attached a simulation routine so that the effect of varied doses could be explored on the amount of bacterial kill over time.

Although we examined a number of single agents, the most promising were PMD, MXF, BDQ and LZD. We looked at all possible combinations of these drugs in 2-drug regimens and in 3 Mtb metabolic states (LPG and APG with Mtb strain H37Rv; NRP using the streptomycin-starved *M. tuberculosis* strain 18b [41,42]). PMD + MXF appeared the most promising 2-drug regimen followed very closely by BDQ as the third agent [11].

Previous work has demonstrated that for treating Mtb, single agent therapy is inferior to dual therapy, which is inferior to 3-drug combinations [43,44]. To identify optimal 3-drug regimens, it was necessary to create a 2-drug mathematical analysis whereby we could calculate a 95% confidence interval around the decline in bacterial burden and also identify whether there was resistance emergence. We had generated a framework for this approach previously [12,15]. We can straightforwardly expand this approach to regimens with larger drug numbers.

In our earlier *in vitro* studies, we found all combination arms of the 2-drug full factorial study design suppressed resistance emergence by day 7 for Mtb H37Rv in log-phase growth. The 3-drug regimen of PMD + MXF + BDQ reduced the quantitative counts of Mtb below the lower bound of the 95% confidence interval, indicating the superiority in bacterial kill of the 3-drug regimen [12]. We also recapitulated this outcome for acid-phase and NRP-phase organisms [13,14].

The three evaluations in three metabolic states indicated that the 3-drug combination of PMD + MXF + BDQ was optimal in the lab. However, we felt it mandatory to perform multiple *in vivo* **prospective validation** experiments in several animal models.

In the BALB/c murine model, the 2-drug regimens were better than single agents and the 3-drug regimen of PMD + MXF + BDQ was the only regimen for which no organisms could be recovered after three months off therapy. This model system was concordant with the assertion derived from our prior *in vitro* analyses that PMD + MXF + BDQ is an optimal 3-drug regimen. In this study cultures of the BDQ+PMD+LZD (BPaL) arm was overgrown by an *Enterobacter* species at all time points, making it impossible to evaluate the effect of this regimen on the killing of Mtb.

Kramnik (C3HeB/FeJ) mice generate pathological features most similar to human TB. Formation of human-like granulomas and other pathology is particularly relevant for drug studies. Consequently, this mouse model system has gained in popularity in recent years [26,28,45,46] In addition to evaluating our proposed optimal regimen of PMD + MXF + BDQ, we also examined the BPaL 3-drug regimen, in which MXF was replaced with LZD, and a 4-drug regimen of PMD + MXF + LZD + BDQ. The 4-drug regimen was added to evaluate whether either 3-drug regimen consisting of PMD and BDQ in combination with MXF or LZD achieved near maximal effect and to assess whether a faster bactericidal rate could be achieved with the 4-drug regimen. The SOC in the studies performed in Kramnik mice was the 4-drug regimen of INH + RIF + PZA + EMB. EMB was added to the SOC for the studies in Kramnik mice since the SOC 3-drug regimen of INH + RIF + PZA did not achieve sterilizing effect in BALB/c mice.

In Kramnik mice, both regimens of PMD + BDQ, with either MXF or LZD as the third active agent, produced similar reductions in Mtb in the lungs and spleens after 1 month of treatment and did not have sterilizing effect. With both regimens, there were no recoverable organisms in the lungs and spleens after 2 months of treatment and both regimens produced sterilizing effects in these organs. The 4-drug SOC regimen had recoverable organisms at the 1 and 2 month treatment time points in the lungs. The SOC regimen resulted in no recoverable Mtb in the spleens after 2 months of treatment. Two months of therapy with the 4-drug SOC in Kramnik mice did not achieve sterilizing effect in either organ (Fig 4A and 4B). Both 3-drug regimens that contained PMD + BDQ, and either MXF or LZD as the third agent, had no recoverable organisms after 2 months of treatment, as did the 4-drug regimen. At 1 month of treatment, there was a small difference between the 2 arms (PMD + MXF + BDQ; PMD + LZD + BDQ) that was not statistically significant (0 versus 0.49 ± 0.84 Log_10_(CFU/g)). Again, samples were plated on charcoal-containing agar.

We found that all three regimens sterilized the spleen by the end of 1 month of therapy (Fig 4B). It is critical to note that all three BDQ-containing arms with only 4 weeks of therapy performed identically at this point to the arms where therapy continued for 8 weeks. However, after therapy cessation in the 4 weeks therapy group, killing did not substantively continue. This may indicate that a minimal duration of therapy is coming into focus.

These data show that PMD + MXF + BDQ was an optimal regimen and that PMD + LZD + BDQ was also an excellent regimen. Both rapidly decreased the bacterial burden to near zero. There was no resistance emergence. Importantly, both murine models prospectively validated the *in vitro* experiments with the conclusions being drawn from mathematical modeling.

This study was not designed to determine how quickly these regimens actually sterilize. While the current data are tantalizing, a different study design is required to formally determine time-to-sterilization. Nonetheless, both murine models prospectively validated the *in vitro* experiments with the conclusions being drawn from mathematical modeling.

Another open question is why these particular 3-drug regimens seem to be optimal. Our hypothesis revolves around BDQ. We examined BDQ concentrations in the lungs of uninfected BALB/c mice (Fig 5) by a MALDI-2 Mass Spectrometry Imaging technique and in infected C3HeB/FeJ (Kramnik) mice in Fig 6. There is a wide distribution of BDQ and its M2 metabolite seen in both. The real issue is how much of these molecules get to the infection site and how long they remain at the primary infection site after therapy is discontinued. Irwin et al demonstrated [28] that in Kramnik mice, the lesion heterogeneity leads to heterogeneity in BDQ concentrations at the effect site following a single oral dose. Ordonez and colleagues [47] looked at labelled BDQ in Kramnik mice and showed that the exit rate from the infection site approximated 12 hours by observation. While this approach cannot differentiate BDQ from its M2 metabolite, this is substantially longer than seen for MXF. Prideaux et al [48] examined MXF in rabbits and found the half-life of exit of this agent from the primary infection site was approximately 5 hours. It is important to note that in Fig 6, BDQ and its active M2 metabolite demonstrate increased signal between hours 1 and 5, while both MXF and LZD demonstrate a major signal decrement over this time frame in both lung and spleen. This indicates that in both animals and humans, there will be substantial amounts of BDQ and metabolite present for long periods of time after therapy cessation. This will have an impact on time-to-sterilization and is likely to also have an impact on resistance emergence for BDQ, as organisms transitioning from NRP-phase to Log-phase late after therapy cessation will be exposed to drug concentrations that will exert selective pressure, but without being able to kill the organisms, setting the stage for a higher probability of resistance emergence.

It was important to examine the drug concentrations for PMD, MXF and BDQ when we evaluated these drug regimens in a third animal model- cynomolgus macaques.

The overall design of this macaque study is shown in Fig 2A. Drug efficacy was measured by three metrics. First, serial FDG PET/CT imaging was performed. FDG uptake is associated with inflammation but also reflects bacterial burden [32–34]. Second, the pathology observed at necropsy was quantitatively assessed. Third, several organs (lung, lymph node, spleen, liver) were cultured to quantify replication-competent Mtb.

Quantitative pathology scores at necropsy poorly differentiated the efficacies of the drug regimens tested. This is likely due to the fact that the pathology associated with Mtb (e.g., lymphadenopathy, lung lesions, etc.) resolves slowly following successful treatment and may be considered a “lagging indicator” of efficacy. However, PET/CT imaging showed clearly that the PMD + MXF + BDQ regimen reduced pulmonary inflammation, as reflected by total FDG activity, more quickly and consistently than the other regimens. This was observed in both cohorts, reinforcing the robustness of this conclusion. The rapidity with which PMD + MXF + BDQ decreased the FDG activity (Fig 2B) may be important. We hypothesize that lowering inflammation may be important in limiting the tissue damage done in response to the Mtb infection. This is important to study in ongoing evaluations, as we would like to identify the regimen that acts most quickly in order to reduce treatment duration.

Since total FDG activity is positively correlated with bacterial load (35–36), the PET/CT imaging data imply that the PMD + MXF + BDQ regimen reduced Mtb burden faster than the other two regimens. However, Mtb bacilli were quantified directly only at necropsy. We found that all three regimens significantly reduced Mtb burden. Only 1 animal in each regimen failed to completely sterilize. The reasons for this are unclear, but may be due to the animal-to-animal heterogeneity common in the macaque model in terms of spatial lesion distribution, disease severity, pharmacokinetics, etc.

The dramatic reduction in bacterial load that resulted from each drug regimen in the macaques was somewhat surprising given that there was little reduction in FDG activity or pathology following either the PMD + MXF + BDQ/LZD + BDQ or the PMD + LZD + BDQ regimen. To determine whether this was an artifact due to drug carryover, we measured the drug concentrations in the tissue homogenates. Even though the antibiotics were discontinued at least 24 hours prior to necropsy, substantial levels of some drugs remained in the tissues (Table 2). A very wide range of concentrations for BDQ and its M2 metabolite were observed. The levels were higher than those measured for LZD, MXF and PMD. Indeed, all MXF samples were below the assay limit of detection. The standard deviations for the tissue drug levels were likewise highest for BDQ and M2 metabolite. This raises the possibility of drug carryover, especially BDQ and its M2 metabolite, as an explanation for the sterility of the tissues plated at necropsy. However, we did plate these samples both on 7H11 agar and on 7H11 agar containing 0.4% activated charcoal. Such charcoal agar has been shown previously to remove anti-tuberculosis drugs from the tissue samples [35]. We also determined how high a drug concentration could be neutralized from a sample with charcoal-infused agar (S1 Fig) and found that 0.4% of activated charcoal added to 7H10 + 10% OADC agar can neutralize at least 26/117 mg/L of BDQ/M2 metabolite, 64 mg/L of LZD, and >128 mg/L of PMD. The efficacy of activated charcoal in neutralizing MXF was not examined because the drug rapidly clears from murine and non-human primate tissues (Fig 4, S1 Fig and Table 2). These studies showed that charcoal-infused agar could remove more than the maximal amounts of BDQ and M2 metabolite found in the tissue homogenates (Table 2). Thus, it is unlikely that the sterility of the macaque tissues is due to drug carryover and strongly implies that all three drug regimens did render the vast majority of the animals sterile. Nonetheless, the PMD + MXF + BDQ regimen appears superior in most quickly reducing lung inflammation by FDG PET/CT. This drug combination may also more quickly lead to sterilization than the other regimens tested, although this was not formally demonstrated here. The macaque study is consistent with the two murine system experiments as noted above. The performance of the other bedaquiline-containing regimens in all three *in vivo* systems is important to note. It points up a limitation of the *in vitro* evaluations. While the PMD/MXF/BDQ regimen was correctly identified *in vitro*, the importance of the BDQ in regimen design was not emphasized. This is because a strength of BDQ only was made manifest in the animal systems. This agent forms depots of the drug and its M2 metabolite, providing long lasting activity. The presence of pathology is also a signal for the drug and metabolite [Fig 6]. We speculate this is the likely explanation behind the superior activity of the 3-drug BDQ regimens. It may also be a risk. Animals, like patients, have true between-subject variance in pharmacokinetic parameter values. In any large patient cohort, a small number of subjects will have a very high clearance for one or more of the agents in a regimen. To decrement the probability of resistance emergence, it is highly important to continue therapy for a sufficient period until all or almost all organisms are eradicated. It would be unwise to rely on the depot BDQ/M2 metabolite alone, as this could result in resistant organisms with a relapse.

In summary, we have developed an algorithm to evaluate new and repurposed agents as they enter the clinical armamentarium so as to identify optimal multi-agent regimens with the dual goal of rapidly sterilizing the infection site (leading to shorter regimen duration) and lowering the probability of resistance emergence. This algorithm was based up on *in vitro* evaluation and cutting edge mathematical modeling including identifying dynamically-linked exposure variables for bacteria kill and resistance suppression, a screening methodology for 2-drug combinations and, most importantly, a high dimensional fully parametric approach that allows determination of significant improvement in regimen (indexed to bacterial kill and resistance suppression) as the regimen goes to 3 agents and beyond. This iterative process identified PMD + MXF + BDQ as a regimen that would likely be optimal. We used BALB/c and C3HeB/FeJ (Kramnik) murine models as well as a cynomolgus macaque model of Mtb infection to test various drug regimens *in vivo*. In each prospective evaluation in these disparate animal models, the PMD + MXF + BDQ regimen was consistently superior to the other regimens tested (or as good as – e.g., substituting LZD for MXF in the Kramnik model).

This multi-pronged approach should be applied to evaluate other regimens. Other important questions to pursue are related to the kinetics of BDQ (and similar compounds) ingress into and egress from the lesions *in vivo*. This would help identify regimens that would be shortest in duration, but also robust enough to prevent relapses in a high proportion of patients. Finally, we have devised methods to extend the modeling approach to allow study of 4-drug regimens. Perhaps a fourth agent may be targeted at a specific metabolic state to shorten therapy. In so doing, we can identify optimal multi-agent regimens to minimize treatment duration and this, in turn, will lower the probability of resistance emergence.

## Supporting information

S1 Fig First agar dilution susceptibility study for BDQ and its M2 metabolite evaluated on 7H10 agar +10% OADC without (A) and with (B) 0.4% activated charcoal. The MICs (mg/L) for BDQ/M2 metabolite were read after 3 weeks of incubation.
(PDF)

S1 Table Pharmacokinetic values of PMD in BALB/c mice (A) and Cynomolgus macaques (B). (PDF)

S2 Table Pharmacokinetic values of MXF in BALB/c mice (A) and Cynomolgus macaques (B). (PDF)

S3 Table Pharmacokinetic values of BDQ in BALB/c mice. (PDF)

S4 Table Pharmacokinetic values of LZD in BALB/c mice (A) and Cynomolgus macaques (B). (PDF)
