# Supplementary material for: Identifying optimal combination regimens for therapy of Mycobacterium tuberculosis with an algorithmic approach: prospective predictions and validations
Source: PLoS One. 2026 Feb 10;21(2):e0324206. doi: 10.1371/journal.pone.0324206 (PMC12890097; doi:10.1371/journal.pone.0324206)

S1 Fig. Quantifying the amount of BDQ, its major metabolite, and PMD that are inactivated by adding 0.4% activated charcoal to TB agar. In the first of three experiments, agar dilution susceptibility studies for BDQ and its M2 metabolite were performed on 7H10 + 10% OADC agar using the fixed ratio of 1:4.5 measured in NHP ELF. The susceptibility studies were performed on TB agar (7H10 + 10% OADC) that was and was not supplemented with 0.4% activated charcoal.

For this study, the MICs for BDQ/M2 metabolite on charcoal-free and charcoal-supplemented agars were 0.03/0.135 and >16/72 mg/L, respectively (Figure S1).

In the second study, the MICs for an extended range of BDQ/M2 metabolite up to 128/576 mg/L in TB agar that was and was not supplemented with 0.4% activated charcoal was evaluated. The MICs on charcoal-free and charcoal-supplemented agars were 0.06/0.27 and 32/144 mg/L, respectively (data not shown).

The third study aimed to more specifically identify the BDQ/M2 MIC when evaluated on charcoal-containing agar. This study used two-fold dilutions of BDQ/M2 metabolite (at the fixed 1:4.5 ratio) from 4/18 to 16/72 mg/L then 2/9 mg/L arithmetic increments of BDQ/M2 metabolite for concentrations between 16/72 and 32/144 mg/L. The MIC for BDQ/M2 compound was 28/126 mg/L on TB agar supplemented with 0.4% activated charcoal and 0.06/0.27 mg/L on TB agar without charcoal. TB agar supplemented with 0.4% activated charcoal with able to inactivate a minimum of 26/117 mg/L of BDQ/M2 metabolite (data not shown).

The same procedure was used to identify the concentrations of LZD that were inactivated by adding 0.4% activated charcoal to TB agar. That study showed that at least 64 mg/L of LZD was absorbed by activated charcoal (data not shown).

S1 Fig. First agar dilution susceptibility study for BDQ and its M2 metabolite evaluated on 7H10 agar + 10% OADC without (A) and with (B) 0.4% activated charcoal. The MICs (mg/L) for BDQ/M2 metabolite after 3 weeks of incubation.

(A) Agar without activated charcoal

(B) Agar with 0.4% Activated Charcoal

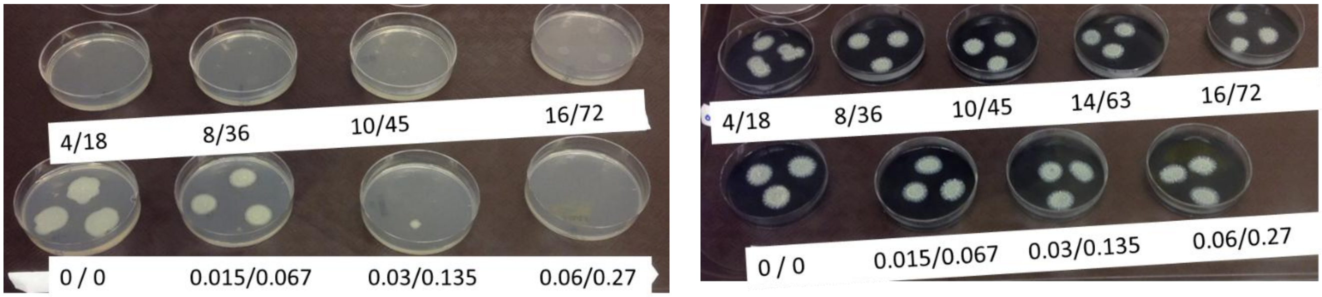

Supplement: S1 Fig — The MICs (mg/L) for BDQ/M2 metabolite were read after 3 weeks of incubation. (PDF) [file pone.0324206.s001.pdf]
