# Supplementary material for: Identifying optimal combination regimens for therapy of Mycobacterium tuberculosis with an algorithmic approach: prospective predictions and validations
Source: PLoS One. 2026 Feb 10;21(2):e0324206. doi: 10.1371/journal.pone.0324206 (PMC12890097; doi:10.1371/journal.pone.0324206)
Supplement: S1 Table — (PDF) [file pone.0324206.s002.pdf]

S1 Table: Pharmacokinetic Values of PMD in BALB/c Mice (A) and Cynomolgus macaques (B).

**Table A**	V	CL	K13	K31	V_{ELF}	Ka
Units	L/kg	L/hr/kg	h^{-1}	h^{-1}	L/kg	h^{-1}
Mean	2.62	0.615	14.9	15.9	3.73	4.60
SD	1.42	0.333	2.57	3.32	2.41	5.76
Median	2.13	0.563	15.4	16.3	3.06	1.00

**Table B**	V	CL	K12	K21	K13	K31	V_{ELF}	Ka	T_{Lag}
Units	L/kg	L/hr/kg	h^{-1}	h^{-1}	h^{-1}	h^{-1}	L/kg	h^{-1}	h
Mean	33.9	15.8	5.29	11.0	11.2	14.6	4.67	0.234	1.47
SD	10.2	15.5	5.38	4.96	3.33	1.94	2.26	0.130	0.948
Median	30.2	10.4	4.60	9.40	12.8	14.9	4.31	0.188	0.646

V=volume of the central compartment; CL=Clearance; K12, K21, K13, K31=intercompartmental transfer rate constants; Velf= Volume of the ELF compartment; Ka=absorption rate constant; Tlag= lagtime to start of absorption.
