# Supplementary material for: Identifying optimal combination regimens for therapy of Mycobacterium tuberculosis with an algorithmic approach: prospective predictions and validations
Source: PLoS One. 2026 Feb 10;21(2):e0324206. doi: 10.1371/journal.pone.0324206 (PMC12890097; doi:10.1371/journal.pone.0324206)
Supplement: S2 Table — (PDF) [file pone.0324206.s003.pdf]

S2 Table: Pharmacokinetic Values of MXF in BALB/c Mice (A) and Cynomolgus macaques (B).

**Table A**	V	CL	Ka
Units	L/kg	L/h/kg	h^{-1}
Mean	1.96	1.53	7.23
SD	3.63	1.28	4.99
Median	1.22	1.26	5.62

**Table B**	V	CL	K12	K21
Units	L	L/hr	h^{-1}	h^{-1}
Mean	6.77	4.80	2.02	1.41
SD	0.474	1.55	0.778	0.291
Median	6.80	4.61	2.00	1.40

V=volume of the central compartment; CL=Clearance; K12, K21 =intercompartmental transfer rate constants.
