# Supplementary material for: Identifying optimal combination regimens for therapy of Mycobacterium tuberculosis with an algorithmic approach: prospective predictions and validations
Source: PLoS One. 2026 Feb 10;21(2):e0324206. doi: 10.1371/journal.pone.0324206 (PMC12890097; doi:10.1371/journal.pone.0324206)
Supplement: S3 Table — (PDF) [file pone.0324206.s004.pdf]

S3 Table. Pharmacokinetic values of BDQ in BALB/c mice.

	V	CL	K13	K31	V_{ELF}	Ka
Units	L/kg	L/hr/kg	h^{-1}	h^{-1}	L/kg	h^{-1}
Mean	16.4	1.57	1.04	1.66	3.79	8.42
SD	7.38	0.447	2.73	4.74	2.06	8.05
Median	17.3	1.45	0.148	0.143	3.25	3.10

V=volume of the central compartment; CL=Clearance; K13, K31=intercompartmental transfer rate constants; Velf= Volume of the ELF compartment; Ka=absorption rate constant.

Note: BDQ PKs in non-human primates were performed by the Global Alliance for TB Drug Development and were not repeated in the current project.
