# Supplementary material for: Identifying optimal combination regimens for therapy of Mycobacterium tuberculosis with an algorithmic approach: prospective predictions and validations
Source: PLoS One. 2026 Feb 10;21(2):e0324206. doi: 10.1371/journal.pone.0324206 (PMC12890097; doi:10.1371/journal.pone.0324206)
Supplement: S4 Table — (PDF) [file pone.0324206.s005.pdf]

S4 Table: Pharmacokinetic Values of LZD in BALB/c Mice (A) and Cynomolgus macaques (B).

**Table A**	V	CL	K13	K31	V_{ELF}	Ka
Units	L/kg	L/hr/kg	h^{-1}	h^{-1}	L/kg	h^{-1}
Mean	1.12	0.520	10.7	9.90	0.992	12.8
SD	0.772	0.124	4.18	0.777	0.369	2.89
Median	1.01	0.490	7.56	9.81	0.864	13.5

**Table B**	V	CL	K12	K21	K13	K31	V_{ELF}	Ka	T_{Lag}
Units	L/kg	L/hr/kg	h^{-1}	h^{-1}	h^{-1}	h^{-1}	L/kg	h^{-1}	h
Mean	3.66	1.58	8.64	13.6	6.96	9.28	0.540	1.16	0.638
SD	1.43	0.747	8.51	5.56	5.25	4.77	0.222	1.57	0.327
Median	3.06	2.24	14.6	14.6	5.60	8.20	0.570	0.498	0.688

V=volume of the central compartment; CL=Clearance; K12, K21, K13, K31=intercompartmental transfer rate constants; Velf= Volume of the ELF compartment; Ka=absorption rate constant; Tlag= lagtime to start of absorption.
